# Strategies towards Producing Non-Polar Dolomite Nanoparticles as Nanofiller for Copolymer Nanocomposite

**DOI:** 10.3390/ijms232012620

**Published:** 2022-10-20

**Authors:** Asfa Amalia Ahmad Fauzi, Azlin Fazlina Osman, Eid M. Alosime, Ismail Ibrahim, Khairul Anwar Abdul Halim, Hanafi Ismail

**Affiliations:** 1 Faculty of Chemical Engineering & Technology, Universiti Malaysia Perlis (UniMAP), Arau 02600, Malaysia; 2 Biomedical and Nanotechnology Research Group, Center of Excellent Geopolymer and Green Technology (CEGeoTech), Universiti Malaysia Perlis (UniMAP), Arau 02600, Malaysia; 3 King Abdulaziz City for Science and Technology (KACST), P.O. Box 6086, Riyadh 11442, Saudi Arabia; 4 School of Materials and Mineral Resources Engineering, Universiti Sains Malaysia, Engineering Campus, Nibong Tebal 14300, Malaysia

**Keywords:** dolomite, particle size reduction, sonication, nanoparticles, surface modification

## Abstract

Poly (ethylene-co-vinyl acetate) (PEVAc) is a copolymer endowed with high elasticity and resilient properties, potentially utilized in various applications. However, the tensile strength of this copolymer is insufficient for use in certain applications that require enough strength to tolerate high external tension or stress. In this study, dolomite was proposed as a nanofiller to reinforce the PEVAc. Raw dolomite was physically and chemically modified in order to improve its mix ability and interfacial adhesion between the PEVAc and dolomite. Initially, the size of dolomite was reduced by combining the ball-milling and tip-sonication methods. SEM, TEM, and XRD were used to characterize the morphology/structure of the raw dolomite and the size-reduced dolomite. Then, a particle size analysis was performed to confirm the average particle size. Our results show that the particle size of dolomite was reduced from 150 µm to 441.4 nm by the physical modification process (size reduction). Based on the TEM analysis, the Feret diameter (d_f_) of the dolomite particles was also reduced from ~112.78 µm to ~139.58 nm only. This physically modified dolomite is referred as dolomite nanoparticles (DNPs), since one or more of its dimensions is less than 100 nm (e.g., thickness and width). To further improve the dolomite and PEVAc matrix interactions, chemical modification of the DNPs were performed by treating the DNPs with stearic acid, forming non-polar dolomite nanoparticles (NP-DNPs). The presence of stearic acid in dolomite was confirmed through FTIR and contact angle analyses. A PEVAc nanocomposite film with NP-NPDs as a nanofiller appeared more homogeneous and exhibited the highest increment in tensile strength and elongation at break. These findings indicated that the combination of ball milling and tip sonication is an efficient method for producing very fine dolomite particles up to the nano-size range, whereas chemical surface modifications improved the compatibility between the dolomite and the copolymer. The combination of these physical and chemical modifications helped to develop a homogeneous copolymer nanocomposite system with improved tensile properties.

## 1. Introduction

A copolymer is a polymer comprising two or more different types of monomers. Examples of copolymers include polymethylmethacrylate, polyethylene tetrafluoroethylene, ethylene-propylene, and styrene butadiene (SBS). These copolymers are utilized in several fields such as packaging, electrical applications, building, and construction [1,2]. Poly (ethylene-co-vinyl acetate) (PEVAc) is a thermoplastic type of copolymer that is derived from the copolymerization process of ethylene (non-polar) and vinyl acetate (polar) monomers. PEVAc exhibits a flexibility similar to elastomeric materials, yet it can be processed similarly to other thermoplastic polymers [3,4]. PEVAc is known to be used in the production of cables, toys, wires, shoes, food packaging, and adhesives [5,6]. In the biomedical field, PEVAc is widely used in catheters, drug delivery systems, and the artificial valves used in heart replacements [7,8]. A PEVAc/MMT nanocomposite has been studied as an insulation material for implantable medical devices [9].

In this research, dolomite was used as a filler for a PEVAc composite/nanocomposite. Dolomite is a mineral-based material. It is a type of sedimentary carbonate rock primarily composed of the dolomite mineral. Dolomite comes from a diverse range of sources; it can be found in lakes or beneath shallow seafloors, and it can also be found in early to late burial settings [10]. Dolomite can be developed through two possible mechanisms, which are direct precipitation and the dolomitization process. In the dolomitization process, calcite is dissolved, supplying Ca^2+^ ions, followed by the precipitation of dolomite from a solution rich in Mg^2+^ ions [11,12,13]. Some dolomites are formed by the replacement of pre-existing limestone during the dolomitization process [11].

Dolomite can be found in several states in Malaysia, with Perlis being one of the largest dolomite producers with several large quarries [14,15,16]. Dolomite found in Perlis is also known as “Batu reput” by the locals and is a source of quality mineral rocks [14]. Perlis’s dolomite is an alkaline earth oxide with a primary composition of CaMg(CO_3_)_2_ and minor impurities such as ferrite and silica. It also contains magnesium ore that is only active when calcined at temperatures above 850 °C and under atmospheric pressure [10]. Dolomite minerals have a similar chemical composition to calcite minerals. Calcite minerals consist of calcium and carbonate (CaCO_3_) layers only, while dolomite minerals consist of three layers made up of alternating calcium (Ca) and magnesium (Mg) layers that are separated by a carbonate (CO_3_) layer. Thus, the structure of dolomite is not exactly in an ordered form, as in calcite, because some of the magnesium may be present in the calcium layers while some of the calcium may be present in the magnesium layers. Dolomite has been used widely in the construction, tar, glass, pharmaceutical, and agricultural industries [17,18,19,20].

Recently, the use of dolomite as a filler in polymer composites has shown an increasing trend [10,21,22,23]. The introduction of dolomite as a filler was intended to enhance the mechanical and thermal properties of polymer composite forms [10,21,22,23]. In addition, the availability of dolomite as a natural resource of the universe gives an advantage to the use of this mineral as a cheap filler, since it can lower the cost of producing products based on polymer composites. Dolomite has the potential to be employed in biomedical applications as a filler in polymer composites because it is non-toxic. However, dolomite cannot be used directly as a filler in polymer composites due to the difficulty of dispersing the raw dolomite in the polymer matrix for the production of a homogeneous polymer composite. Dolomite has hydrophilic properties, while most polymers are hydrophobic. Thus, they are not compatible with each other. Besides, raw dolomite usually contains large particles. A large (micron-sized) filler would reduce the interactions between the filler and the polymer. In addition, the agglomeration of the filler in the polymeric matrices might occur, creating a polymer composite with poor performance, especially with regards to its mechanical and thermal properties [24,25,26].

In this research, the combination of physical and chemical modification techniques was employed in order to produce non-polar dolomite nanoparticles (NP-DNPs). Firstly, the dolomite nanoparticles (DNPs) were prepared using physical modification (size reduction), specifically via the ball-milling and tip-sonication processes. High-intensity ball milling is a top-down grinding process commonly used to generate fine particles. The planetary ball-milling method was used in this study. Large solid particles were mechanically broken down into smaller solid particles during planetary ball milling without changing their chemical structure [27]. The mechanical disintegration of solid particles occurs when the balls are moved vigorously and transform the particles in the jar. A variety of parameters influence the final product size, such as the rotation speed, time, ball-to-powder ratio, ball amount, ball type, and ball diameter [27]. In general, the particle size becomes smaller as the speed and duration increase. The high-speed rotation of the jar and revolution of the disk make the balls move strongly and violently, leading to the fine grinding of the product due to the generation of a large ball-impact energy [10,27]. Several researchers have reported the use of a planetary ball mill to reduce the particle size of dolomite to a submicron size [17,28]. However, none of them successfully reduced the dolomite particle size down to the nano-size range. Furthermore, they used raw dolomite from different sources and with different sizes; therefore, it is not appropriate to just follow the milling parameters used by them in our study. Therefore, in this research, we investigated the best ball-milling parameters to produce the finest dolomite particles. Next, ultrasonication was employed to further reduce the particle size and to de-agglomerate the possible agglomerated particles after the ball-milling process. Basically, there are two types of ultrasonication methods. They are the tip or probe-type sonicator and the bath-type sonicator. The tip sonicator is known to be more efficient at de-agglomerating particles when compared to the bath-type sonicator. This is due to the high intensity of sonication that is applied directly to the sample, imparting more concentrated energy to the sample. On the other hand, the bath sonicator isolates the sample from the energy source and requires a greater energy input than the tip sonicator because the entire water bath is energized [29]. Ahmad Fauzi et al. and Lim et al. proved that tip sonication is an effective way to break and de-agglomerate mineral-based particles [10,17,30,31]. However, they did not obtain nano-sized dolomite due to an incomplete investigation of the tip-sonication parameters that can result in the finest particles of dolomite. In this current research, we attempted to fill this research gap by investigating the best parameters for producing nano-sized dolomite (dolomite nanoparticles).

After the physical modification was completed, the chemical modification process was performed. The DNPs were treated with stearic acid. Stearic acid is a natural saturated fatty acid found in the combined form of animal and vegetable fat. It was used as a surface modifier, as it has an alkyl chain group that can change the polarity of dolomite. According to Cao et al., stearic acid is absorbed onto the surface of dolomite via a chemical reaction between stearic acid’s “head” and a calcium cation, thus creating a hydrophobic monolayer film on the surface of the dolomite [32]. In addition, most research states that using stearic acid is a green approach, as it is non-toxic. Thus, it is suitable to be used as a filler. The physically and chemically treated NP-DNPs were employed as a nanofiller for the PEVAc copolymer nanocomposite to improve the homogeneity of the dolomite/PEVAc mixture, and thus, improve the tensile properties of the resultant copolymer/dolomite film.

## 2. Results and Discussion

The size reduction of dolomite particles from their raw form was first analyzed using a scanning electron microscope (SEM). Then, the samples with the most significant size reduction were further analyzed using transmission electron microscopy (TEM), which enabled the imaging of an individual particle (separated particle) of dolomite. Then, a particle size analysis was performed in order to confirm the size of the dolomite.

Next, raw dolomite (RD), dolomite nanoparticles (DNPs), and non-polar dolomite nanoparticles (NP-DNPs) were characterized by X-ray diffraction (XRD), Fourier-transform infrared spectroscopy (FTIR), and the contact angle. RD, DNPs, and NP-DNPs were used as fillers/nanofillers in the PEVAc as a copolymer matrix. The resultant composite/nanocomposite samples were subjected to tensile analysis and their fractured surfaces were analyzed using an SEM.

### 2.1. Characterization of Raw Dolomite, Milled Dolomite, and Dolomite Nanoparticles (DNPs)

#### 2.1.1. Scanning Electron Microscope (SEM)

**Figure 1:**
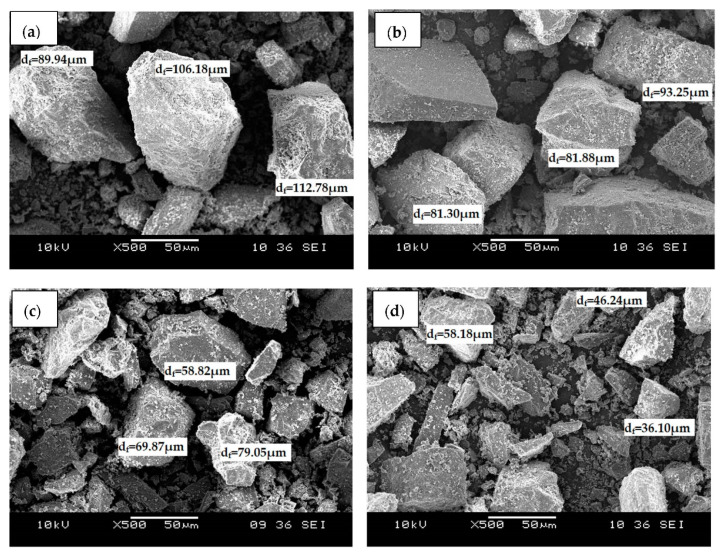
SEM micrographs of (**a**) raw dolomite (RD), (**b**) D_300_, (c) D_400_, and (**d**) D_500_ at ×500 magnification.

Figure 1 shows the SEM micrographs of (a) raw dolomite (RD) and dolomite that has been ball-milled at a speed of (b) 300 rpm (D_300_), (c) 400 rpm (D_400_), and (d) 500 rpm (D_500_). The micrographs revealed that the dolomite had an irregular shape and rhombohedral structure. The shape and structure of the dolomite appeared identical in all images, even when the dolomite was ball-milled with the highest speed, which was 500 rpm. Originally, the dolomite powder was supplied with a particle size of less than 150 µm as shown in Figure 1a. However, as it was ball-milled, the size of the dolomite was reduced. Apparently, finer dolomite particles can be spotted in Figure 1c,d, which correspond to the dolomite that was ball-milled at 400 rpm and 500 rpm, respectively. The dolomite that was ball-milled with a speed of 300 rpm and 400 rpm were reduced to a Feret diameter (d_f_) of 81 µm to 93 µm and 58 µm to 79 µm, respectively. Meanwhile, the size of the dolomite that was ball-milled at a speed of 500 rpm was further reduced to an average length of 36 µm to 58 µm. This indicates that the higher the rotational speed, the smaller the size of the milled materials [33,34,35]. The reduction in the dolomite’s particle size could be attributed to the possible friction between the steel balls and the sample or between the particles within the sample, as well as contact between the sample and the walls of the jar. This possible friction could have led to the rupture of joints, cleavage, and massive breakage of the constituent particles [27,36,37,38]. However, milling times that are too lengthy may result in the particle size reduction reaching its limit. Nik Nur Azza et al. discovered that milling for 5, 10, and 20 h produced particles with not much size difference, suggesting that longer milling times did not necessarily reduce the dolomite’s particle size [39]. This could be the result of particle agglomeration. This agglomeration may cause a plateau effect on the size of the dolomite particles when longer milling times and higher milling speeds are used [35]. For this reason, the milling time in this study was maintained at 6 h and the maximum speed used was 500 rpm.

**Figure 2:**
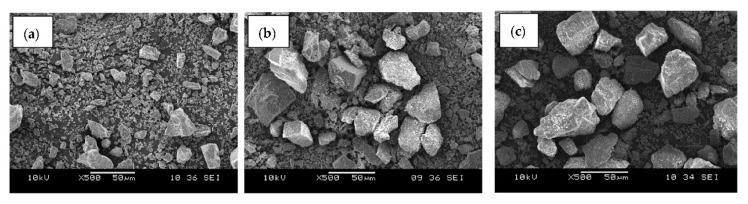
SEM images of dolomite that has been tip-ultrasonicated for (**a**) 2 h, (**b**) 3 h, and (**c**) 5 h at ×500 magnification.

Figure 2 presents SEM micrographs of dolomite that has been tip-ultrasonicated with 30% amplitude (constant) and various durations of time; (a) 2 h, (b) 3 h, and (c) 5 h. Figure 2a shows smaller dolomite particles and the particle size distribution when compared to the ones in Figure 2b,c, suggesting that the size of the dolomite was reduced when it was ultrasonicated for 2 h. The sonic wave traveled into the particles through the liquid medium during the ultrasonication process. As a result, it created alternating high- and low-pressure cycles. Then, the high-intensity sonic waves created a large number of microbubbles during the low-pressure cycles, which later collapsed during the high-pressure cycles in a very short time. This process is known as ultrasonic cavitation. These processes cause a high local temperature, high-speed impinging liquid jets, and strong hydrodynamic shear forces. Through these effects, particles were broken down, disintegrated, and de-agglomerated [30,40,41]. Thus, the particle size was significantly reduced.

Figure 2b,c reveal that the size of the dolomite became larger when a longer tip-ultrasonication time was used (3 h and 5 h). This demonstrates that the duration of ultrasonication influenced the particle size. It has been proven that the particle size decreases as the duration of ultrasonication increases [42]. However, according to several other studies, the particle size increases over a longer time due to the re-agglomeration of unstable particles [30,43]. This finding is in line with the results of Ali et al., who discovered an increase in vermiculite particle size as the ultrasonicated time increased due to particle aggregation [44]. Nguyen et al. also claimed that after a particular duration of ultrasonication, the particle size increases because the particles may regroup back together while still having a low driving energy to de-agglomerate [43]. According to Afzal et al., stable particles in solution can be created if the ultrasonication time is sufficient, resulting in fewer agglomerated particles [29]. However, particle re-agglomeration may occur if the processing time exceeds the optimal limit. In our case, the ideal ultrasonication time to best reduce the dolomite particle size was 2 h, which means that extending this length may result in a reversal of the impact.

**Figure 3:**
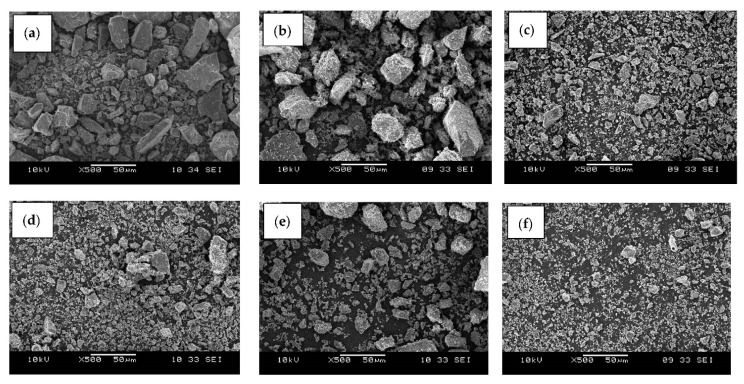
SEM images of dolomite that has been ball-milled for 500 rpm and ultrasonicated for 2 h with (**a**) 30% amplitude and 1 repetition (D_301X_), (**b**) 40% amplitude and 2 repetitions (D_402X_), (**c**) 40% amplitude and 3 repetitions (D_403X_), (**d**) 50% amplitude and 2 repetitions (D_502X_), (**e**) 50% amplitude and 3 repetitions (D_503X_), and (**f**) 50% amplitude and 10 repetitions (D_510X_).

Figure 3 reveals SEM micrographs of dolomite that has been ball-milled at a speed of 500 rpm and tip-ultrasonicated for 2 h (constant), but with a varying amplitude and repetition. As shown in Figure 3c–f, the particle size became finer and the size distribution of the dolomite became smaller as the amplitude and repetition parameters of the ultrasonication increased. The finest dolomite particles with the smallest size distribution are shown in Figure 3f, wherein the dolomite was ultrasonicated with an amplitude of 50% and a repetition of 10 times. The exact size of this dolomite is revealed in the TEM and particle analysis sections.

#### 2.1.2. Transmission Electron Microscopy (TEM)

**Figure 4:**
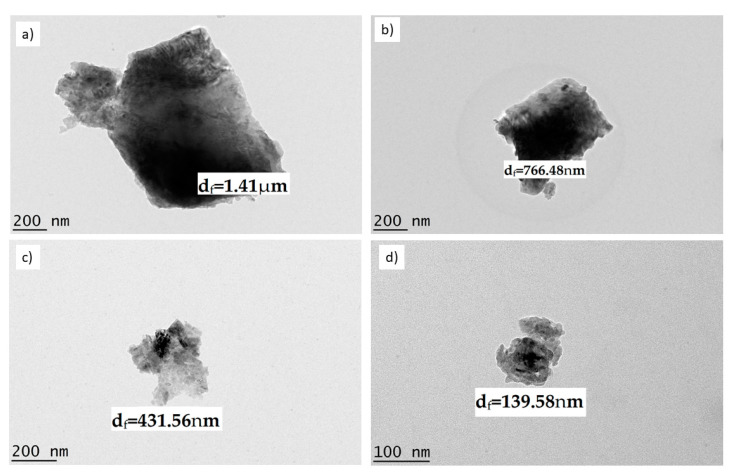
TEM images of dolomite that has been ball-milled at 500 rpm and ultrasonicated for 2 h with (**a**) 30% amplitude and 1 repetition (D_301X_), (**b**) 50% amplitude and 2 repetitions (D_502X_), (**c**) 50% amplitude and 3 repetitions (D_503X_), and (**d**) 50% amplitude and 10 repetitions (D_510X_).

Raw dolomite with micron-sized particles can be easily imaged using an SEM. Based on Figure 1a, it is known that an individual dolomite particle’s size can be as large as ~112.78 µm (d_f_). However, when the particle size of dolomite has been significantly reduced to the nano-size range, it is not appropriate to employ an SEM as an imaging technique for capturing the image of an individual particle of dolomite. Therefore, TEM was employed in this case. Figure 4a–d show the TEM images of D_301x_, D_502X_, D_503X_, and D_510x_. The d_f_ values for D_301x_ and D_502X_ were ~1.41 µm and ~766.48 nm, respectively. A reduction in particle size could be significantly observed when higher amplitudes and more repetitions were applied. For instance, D_503X_ had a Feret diameter (d_f_) of 431.56 nm, while D_510x_ had a d_f_ of 139.38 nm. These results proved that the size of the dolomite particles underwent the greatest reduction when a 50% amplitude and a repetition of 10 times was applied. In another study, the size of vaterite also decreased with an increasing amplitude of sonication [45]. Nguyen et al. also proposed that the particle size decreases with increasing frequency and amplitude [43]. This is because the higher the amplitude, the bigger the force given to the particles in order to break them. Thus, a greater reduction in size can be noticed.

**Figure 5:**
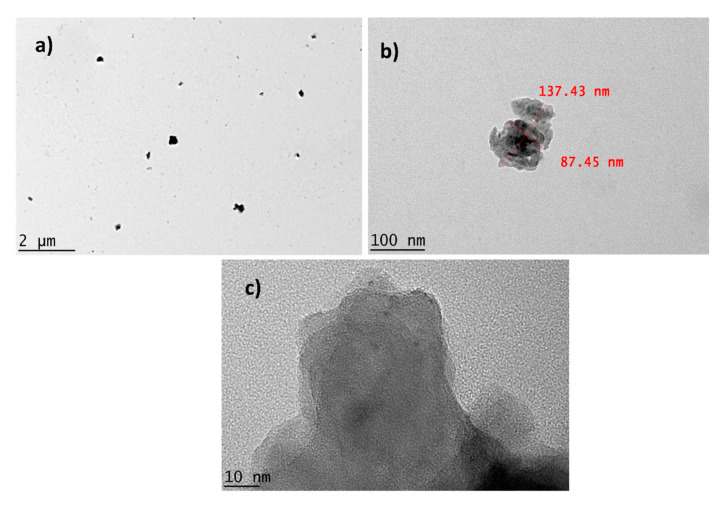
Images of dolomite nanoparticles (DNPs): (**a**) at low magnification, (**b**) at high magnification (with the measured length and width), and (**c**) platy/sheet-like particles of DNPs with a very small thickness (less than 100 nm).

As shown in Figure 4c,d, the shape of D_503X_ and D_510x_ were less defined (with no sharp edges) when compared to that of D_301x_ and D_502x_. As mentioned earlier, when a higher amplitude was applied, a bigger impact was applied to the dolomite particles and thus, this ruptured and diminished the trigonal rhombohedral structure (carbonate group structure). Figure 5 illustrates images of the D_510x_ sample (DNPs) at low and high magnifications. The low-magnification image shows the presence of very fine particles and some bigger (agglomerated) particles. The high-magnification image of individual (de-agglomerated) particles shows one dimension of less than 100 nm (87.45 nm in width). The close-up image of the DNPs shows that they possessed platy shapes with a very small thickness (a few nanometers only). Nevertheless, the results proved that by employing both ball-milling and ultrasonication methods, the size of dolomite can be reduced down to the nano-size range.

#### 2.1.3. Particle Size Analysis

**Figure 6:**
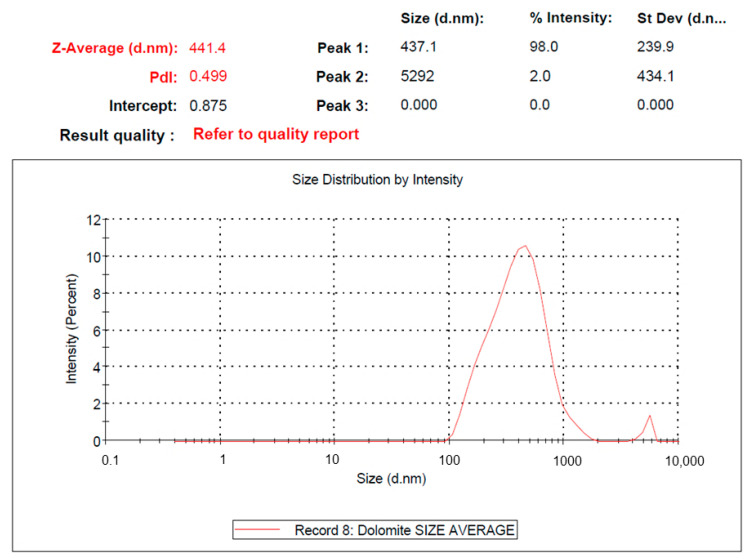
Particle size analysis of the dolomite nanoparticles (DNPs) obtained through milling and tip-sonication processes.

The average particle size of the dolomite nanoparticles (D_510X_ or DNPs) was examined using a particle size analysis (Malvern Zetasizer). The results are presented in Figure 6, showing that the average particle size of dolomite that has been ball-milled and tip-ultrasonicated at 50% amplitude for 2 h and over 10 times is around 441.4 nm (in length). It is worth mentioning that this particle size analysis can only measure the diameter (length) of the nano-dolomite, since it exists in a platy and irregular shape, and not a uniform shape (see Figure 4 and Figure 5). Agglomeration and the overlapping of several numbers of nanoparticles can occur, thus resulting in a greater measurement of the length as compared to the individual (separated) nanoparticles. Nevertheless, this analysis proved that the particle size of the nano-dolomite obtained through the combination of milling and tip-ultrasonication techniques was still in the nanometer range. As with other types of mineral fillers such as nanoclay, the thickness of the platy dolomite nanoparticles could be a few nanometers only (less than 100 nm) (see TEM image in Figure 5c). Unfortunately, the thickness of the dolomite nanoparticles could not be measured using this particle size analyzer equipment.

Based on the SEM, TEM, and particle size analyses, the DNPs could be defined as nanofillers, where at least one of their dimensions was less than 100 nm. The nanoparticles appeared in the form of sheets of one to a few nanometers thick and hundreds of nanometers long. Thus, these Perlis dolomite nanoparticles could be used in the production of polymer nanocomposites.

**Figure 7:**
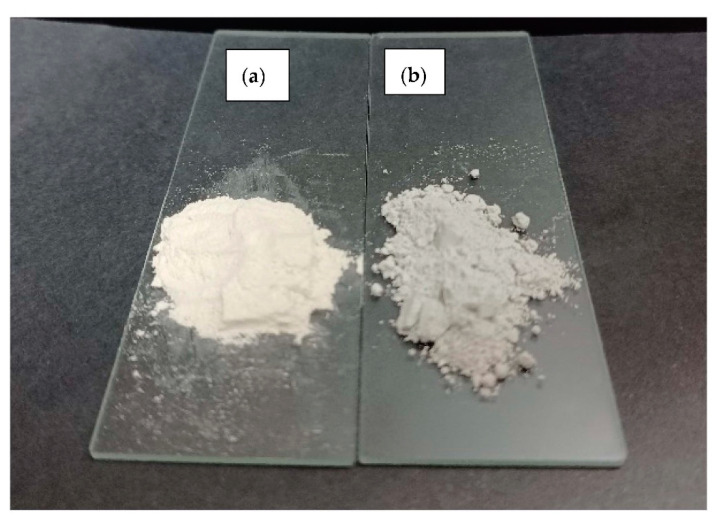
Photos of (**a**) raw dolomite (RD) and (**b**) dolomite nanoparticles (DNPs).

Upon size reduction, the color of the dolomite also changed. Figure 7 shows that originally, the RD had a beige color, but as its particle size was reduced to the nanometer range, the color changed to greyish.

### 2.2. Characterization of Raw Dolomite (RD), Dolomite Nanoparticles (DNPs), and Non-Polar Dolomite Nanoparticles (NP-DNPs)

In the following sections, the discussions are focused on raw dolomite (RD), dolomite nanoparticles (DNPs), and non-polar dolomite nanoparticles (NP-DNPs), as these materials were utilized as a filler/nanofiller for the production of copolymer composites/nanocomposites. The samples were characterized and compared based on XRD, FTIR, and contact angle analyses. The non-polar dolomite nanoparticles (NP-DNPs) were the particles that were chemically treated to obtain a non-polar (organophilic) nanofiller.

#### 2.2.1. X-ray Diffraction Pattern of Raw Dolomite (RD), Dolomite Nanoparticles (DNPs), and Non-Polar Dolomite Nanoparticles (NP-DNPs)

**Figure 8:**
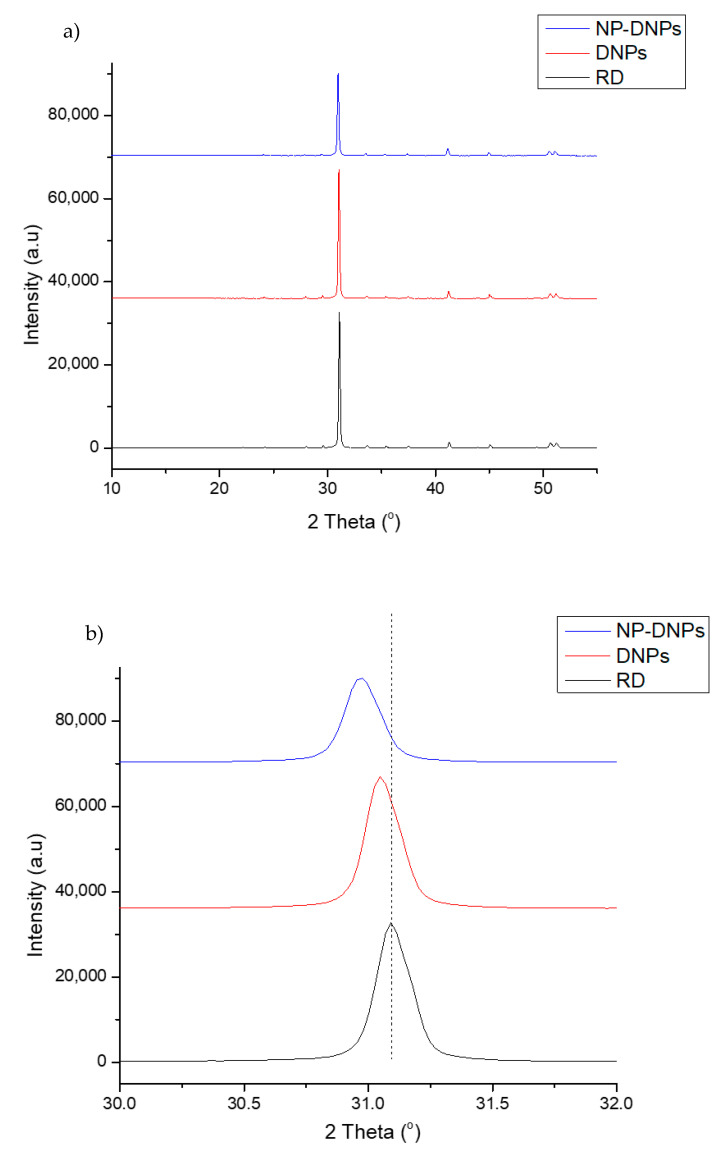
X-ray diffraction pattern of raw dolomite (RD), dolomite nanoparticles (DNPs), and non-polar dolomite nanoparticles (NP-DNPs) that focus on the 2 theta ranges between (**a**) 10° and 55° and (**b**) 30° and 32°.

Figure 8a shows the XRD diffractogram of RD, DNPs, and NP-DNPs. All samples exhibited strong peaks at 2ϴ = 31.04° and 2ϴ = 30.96°, which indicated the presence of dolomite as a prominent mineral (PDF No: 01-075-1656). There was also a quartz mineral peak present at 2ϴ = 27.99° (PDF No: 01-079-1913) and calcite mineral peaks present at 2ϴ = 29.51° and 39.30° (PDF No: 01-072-1650). Other researchers have also reported similar results where the prominent peak of the dolomite mineral was at 2ϴ = ~30° [46,47,48,49].

Figure 8b focuses on the prominent peak of the dolomite mineral in RD, DNPs, and NP-DNPs. The DNPs and NP-DNPs peaks became broader and less intense, and shifted towards a lower angle, when compared to that of RD. According to Tengku Mustafa et al., the ground product had a broader and less intense effect than raw dolomite due to the formation of amorphous materials [15]. This is because the crystalline structure of the materials was distorted and disordered during the mechanical milling process. Besides milling, the harsh ultrasonication treatment also destroyed the crystalline structure of dolomite. The ultrafine and nano-sized particles also produced a wider peak compared to that of the micron-sized particles [48,50]. This is linked to the previous discussion, where the DNPs and NP-DNPs were in the nanometer-size range (based on the TEM and particle size analyses).

The crystallite size and crystallinity of the RD, DNPs, and NP-DNPs are shown in Table 1. When compared to the crystallinity of RD (83.29%), the crystallinity of both DNPs and NP-DNPs showed lower values (79.07% and 80.06%, respectively). As the particle size decreased, the crystallite size was also reduced to 57.76 nm (DNPs) and 57.12 nm (NP-DNPs). As previously stated, the peak intensity of both samples became less intense due to the samples becoming more amorphous as the crystalline structure was distorted during the milling process. The friction between the balls and the sample, the sample and sample, and the wall and the sample disrupted the ordered crystal structure of the dolomite [50]. The reduction in crystallite size and crystallinity were due to the same reason. In addition, changes in dolomite’s morphology (shape/size) caused by ball milling and tip ultrasonication were also noticed in the TEM images.

#### 2.2.2. Fourier-Transform Infrared Spectroscopy (FTIR) Analysis

**Figure 9:**
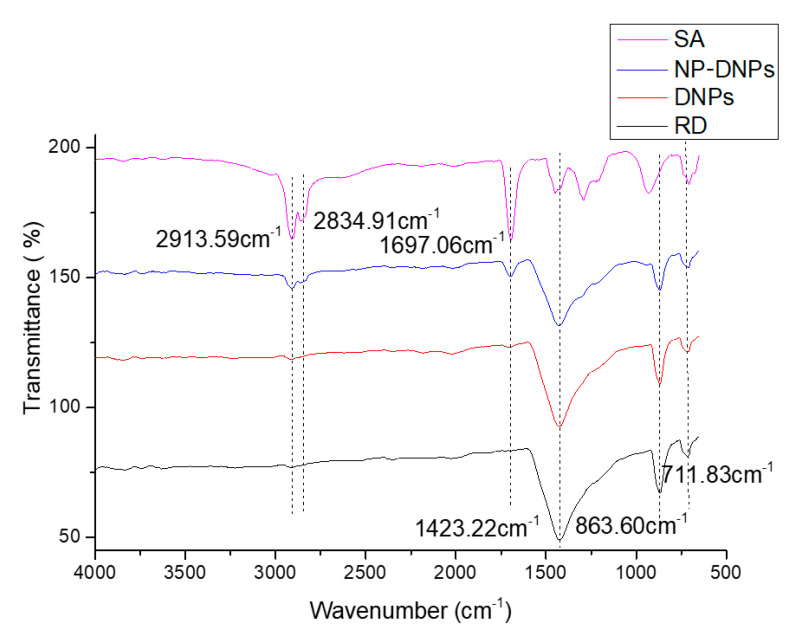
FTIR spectra of raw dolomite (RD), dolomite nanoparticles (DNPs), non-polar dolomite nanoparticles (NP-DNPs), and stearic acid (SA).

A Fourier-transform infrared spectroscopy (FTIR) analysis was performed to identify the functional groups present in dolomite (before and after the stearic acid treatment). Figure 9 shows the FTIR spectra of RD, DNPs, and NP-DNPs.

The FTIR spectra revealed the appearance of three main peaks from the RD sample. The first peak was present at 1423.22 cm^−1^, which was attributed to the asymmetric stretching vibration of the (CO_3_)^2−^ group. Other strong peaks present at 863.60 cm^−1^ and 711.83 cm^−1^ were attributed to the out-of-plane asymmetric and in-plane symmetric bending vibration mode of the O-C-O bond in the (CO_3_)^2−^ of dolomite [51,52,53]. The DNPs and NP-DNPs also exhibited similar peaks. Our findings are in agreement with previously published data [53,54,55,56]. According to Ji et al. and Gunasekaran et al., the peaks present at 1420 cm^−1^, 873 cm^−1^, and 719 cm^−1^ are associated with dolomite [57,58].

In the stearic acid spectrum (SA), there were strong peaks present at 2913.59 cm^−1^ and 2834.91 cm^−1^. These peaks indicated the presence of asymmetric and symmetric stretching vibrations of the aliphatic C-H group, respectively. This is agreement with the findings of Lim et al. and Chen et al. [17,59]. There was also another peak appearing at 1697.06 cm^−1^, which indicated the presence of symmetric stretching vibrations of the C=O group from the stearic acid.

In the NP-DNP spectrum, the main peaks of stearic acid, which was the aliphatic group, were present at ~2900 cm^−1^ and ~2800 cm^−1^. Another peak was also observed at ~1700 cm^−1^, which corresponded to the presence of carboxylic acid (COOH) [60]. As these typical peaks of stearic acid were present in the NP-DNP spectrum, we can confirm that the chemical surface treatment of dolomite was successful. This is in agreement with other findings involving a stearic acid treatment applied to mineral fillers, where similar peaks were present [54,59].

#### 2.2.3. Contact Angle Measurements

**Figure 10:**
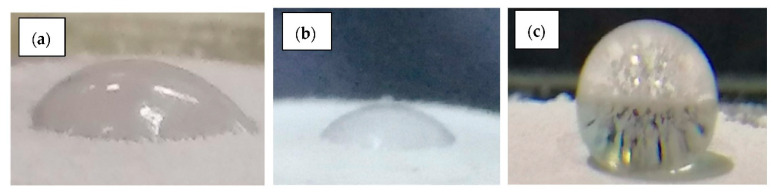
Images of a water droplet on the surface of (**a**) raw dolomite (RD), (**b**) dolomite nanoparticles (DNPs), and (**c**) non-polar dolomite nanoparticles (NP-DNPs).

The contact angle of dolomite was measured to study the wetting state of dolomite before and after the chemical modification with stearic acid. Figure 10 reveals the image of a water droplet on the surface of dolomite, while Table 2 shows the average contact angle. The RD and DNPs exhibited a high wettability from the water droplet with contact angles of 53.36° and 57.07°, respectively. On the other hand, the NP-DNPs showed a low wettability with a contact angle of 140.18° (see Figure 10c). These results further confirmed the presence of stearic acid on the dolomite. In the FTIR analysis, the presence of the aliphatic group was realized in the NP-DNPs spectra. This aliphatic group provided dolomite with non-polar properties. According to Cao et al., during the treatment, the aliphatic group of the stearic acid can be absorbed onto the surface of dolomite via a chemical reaction between the head part of stearic acid and a calcium cation [32], thus creating a monolayered film of a hydrophobic layer covering the dolomite surface. Consequently, the dolomite’s surface changed from polar to non-polar (organophilic).

### 2.3. Characterization of PEVAc Nanocomposite

#### 2.3.1. Tensile Strength, Elongation at Break, and Modulus of Elasticity for PEVAc Nanocomposites

**Figure 11:**
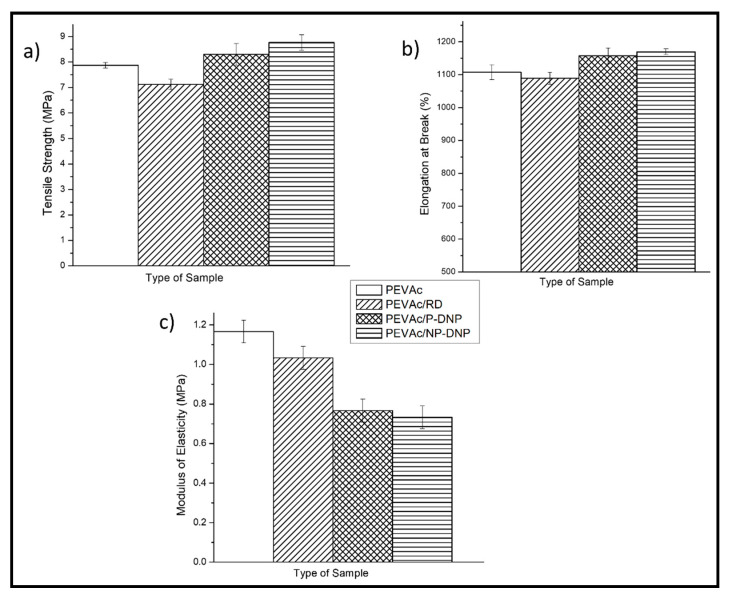
Tensile properties of PEVAc containing raw dolomite (RD), dolomite nanoparticles (DNPs), or non-polar dolomite nanoparticles (NP-DNPs): (**a**) tensile strength, (**b**) elongation at break, and (**c**) modulus of elasticity.

**Figure 12:**
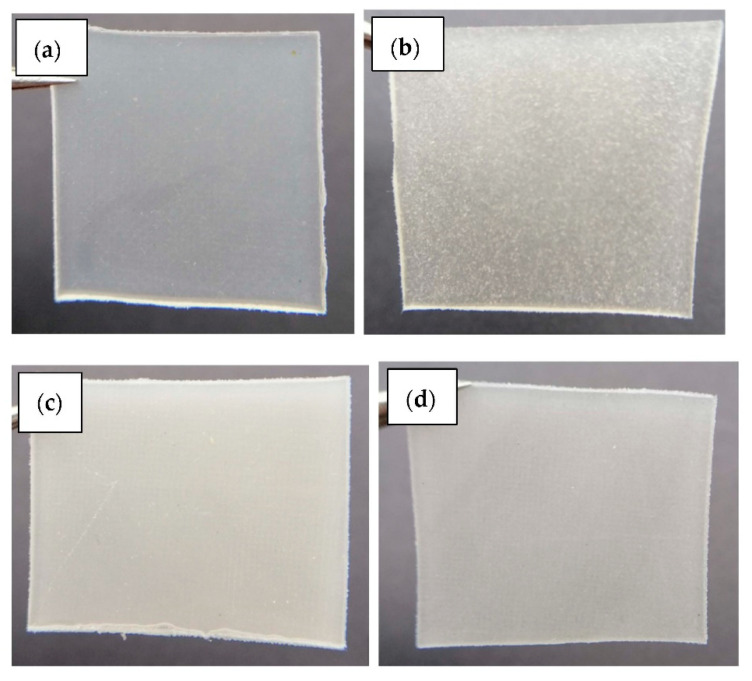
Images of (**a**) PEVAc, (**b**) PEVAc/RD, (**c**) PEVAc/DNP, and (**d**) PEVAc/NP-DNP.

**Figure 13:**
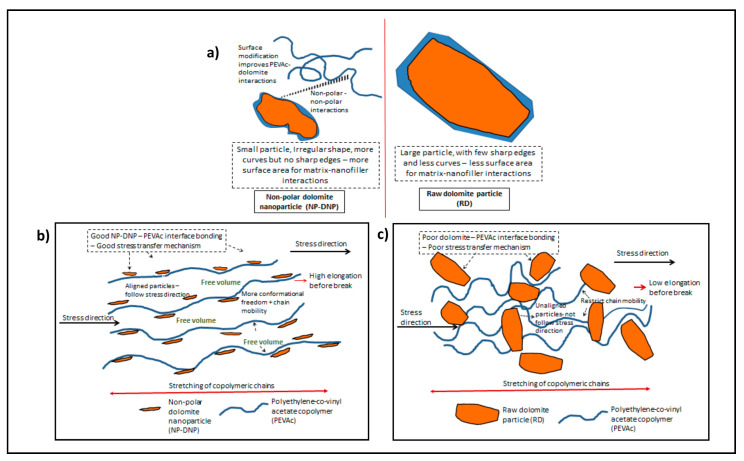
A comparison of (**a**) particle morphology and surface properties of non-polar dolomite nanoparticles (NP-DNPs) and raw dolomite (RD), (**b**) NP-DNP nanofiller effect on the strain behavior of the PEVAc copolymer, and (**c**) RD filler effect on the strain behavior of the PEVAc copolymer.

Figure 11 compares the tensile strength, the elongation at break, and the modulus of elasticity for the PEVAc/RD, PEVAc/DNP, and PEVAc/NP-DNP, while Table 3 summarizes their tensile properties. Initially, the virgin PEVAc exhibited a tensile strength of 7.87 MPa, but with the addition of raw dolomite, the tensile strength decreased by 9.53% to 7.12 MPa. This decrement was due to the addition of the bulky particles of raw dolomite that were poorly dispersed in the PEVAc matrix, thus creating a non-homogeneous PEVAc composite. The direct photo of the PEVAc/RD composite in Figure 12b reveals the inhomogeneity of the PEVAc/RD mixture. The distribution of the agglomerated RD particles can be clearly seen in the transparent film of the PEVAc. On the contrary, the tensile strength of the PEVAc composite with DNPs or NP-DNPs as a filler was found to increase to 8.30 MPa and 8.76 MPa, respectively (increment of 5.46% and 11.31%, respectively). The improvement in the tensile strength was due to the well-dispersed and uniformly distributed DNP and NP-DNP nanofillers in the PEVAc matrix. Figure 12c,d reveal homogeneous films of DNPs and NP-DNPs, with no signs of particle agglomeration. A nano-sized filler is known to provide better filler–polymer interactions due to its larger surface area. Furthermore, small particles are more mobile and more easily dispersed throughout the matrix. As illustrated in Figure 13a, the NP-DNPs contained much finer particles with an irregular shape and more curves. These characteristics contributed to a high-surface-area nanofiller that could more easily disperse through and interact with the PEVAc matrix. Furthermore, the non-polar surface characteristic of the PEVAc caused better nanofiller–matrix interactions. On the contrary, RD had much bigger particles, with fewer curves and few sharp edges, thus resulting in a lower-surface-area filler. The RD particles interacted poorly with the PEVAc matrix.

The results also revealed that the tensile strength of the PEVAc/NP-DNP nanocomposite was higher than the PEVAc/DNP nanocomposite. This was due to the fact that the NP-DNP nanofiller was more compatible with the PEVAc copolymer, as it possessed organophilic surface properties. As mentioned earlier, PEVAc contains two different phases with different polarities (non-polar polyethylene chains versus polar poly (vinyl acetate) chains). However, the non-polar molecule content is higher than the polar molecule content (polyethylene phase = 75%, poly (vinyl acetate) phase = 25%). Thus, having NP-DNPs as a nanofiller for the nanocomposite will result in better dolomite–PEVAc interactions. Subsequently, the tensile strength of the copolymer increased. This finding is also in agreement with the one reported by Lim et al. [17].

The elongation at break also showed a similar trend, where the value decreased with the addition of raw dolomite and increased with the addition of DNPs or NP-DNPs. The elongation at break of the PEVAc/RD decreased by 16.6%, changing from 1107.73% to 1089.33%. As illustrated in Figure 13b,c, the presence of large particles of raw dolomite increased the stiffness of the PEVAc by restricting the molecular motion of the copolymeric chains. In addition, the unaligned particles, which do not follow the direction of stress, would have hindered the stretching of the copolymeric chains to a greater extent. Consequently, the matrix became brittle and more easily fractured under the tensile load. On the other hand, the elongation at break of the PEVAc/DNP and PEVAc/NP-DNP increased by 4.5% and 5.6%, respectively. This suggests that the smaller-sized dolomite fillers did not restrict the movement of the PEVAc molecular chains, but moved and aligned together with the copolymeric chains [31,60]. The homogeneous distribution of the NP-DNP nanofiller also allowed the stretching of the matrix to a greater extent before it broke under the tensile load. This is because when this small-sized filler was embedded into the matrix phase, it allowed the molecular motion of the copolymer chains, and at the same time it helped to transfer the stress throughout the matrix phase. This allowed the energy absorption mechanism during tensile deformation, allowing a greater matrix elongation before failure. That is why the elongation at break of the PEVA nanocomposite containing the NP-DNP nanofiller was greater than those of the the virgin PEVAc and the PEVA/RD composite. Our results are in agreement with the findings of Ridhwan et al. and Lim et al., where a smaller-sized and chemically-treated filler improved the elongation-at-break properties of polymeric matrices [15,17].

On the contrary, the modulus of elasticity values for the PEVAc decreased with the addition of both the DNP and NP-DNP nanofillers. The PEVAc/RD showed a greater modulus of elasticity value, which was 1.03 MPa, when compared to the PEVAc/DNP and PEVAc/NP-DNP, which had modulus elasticity values of 0.77 MPa and 0.73 MPa, respectively. A low modulus of elasticity indicates an elastic material. One of the reasons why PEVAc still possessed elastic properties even though dolomite was added might be due to the nano-sized filler, which can promote chain relaxation in the stress concentration region. It is understood that a nano-sized filler is more mobile and can freely move within a matrix. Therefore, with a nano-sized filler, the PEVAc composite could deform elastically. On the contrary, raw dolomite caused the PEVAc composite to become stiffer. The stiffness of the large particles of dolomite restricted the chain mobility of the copolymer, causing a higher modulus of elasticity of the matrix [21].

#### 2.3.2. Scanning Electron Microscope Analysis of Fracture Surface of PEVAc, PEVAc/RD, PEVAc/DNP, and PEVAc/NP-DNP

**Figure 14:**
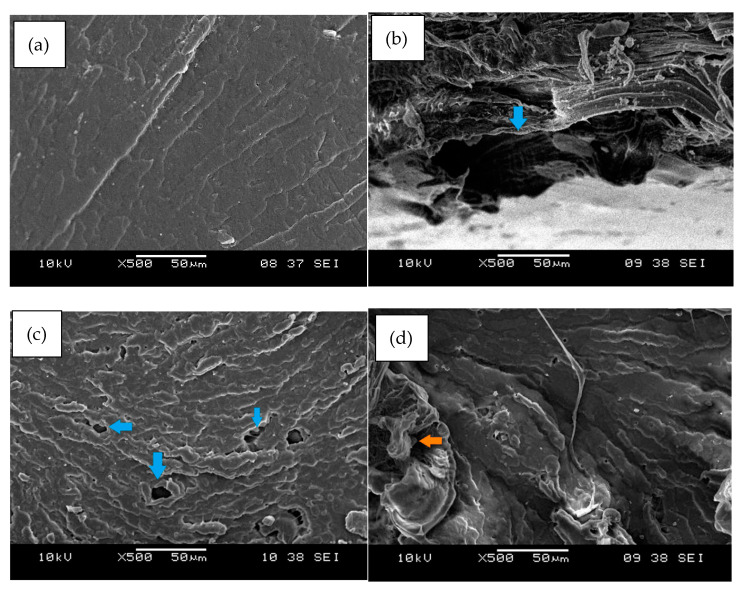
Fracture surface morphology of (**a**) PEVAc, (**b**) PEVAc/RD (The arrow shows the large void on the surface of the matrix (**c**) PEVAc/DNP (The arrows show tiny voids on the surface of the matrix), and (**d**) PEVAc/NP-DNP (The arrow shows the embedded dolomite in the matrix).

Figure 14 reveals the fractured surface morphology of (a) PEVAc, (b) PEVAc/RD, (c) PEVAc/DNP, and (d) PEVAc/NP-DNP. The virgin PEVAc possessed a smooth fractured surface, while the PEVAc/RD showed a rough surface. This was due to the poor dolomite dispersion in the PEVAc matrix. A large void could also be spotted due to the filler pulling out upon stretching (see Figure 14b), which was caused by the weak interfacial bonding of the raw dolomite filler with the matrix. A large filler size has a lower surface area, thus weakening the interfacial adhesion between the dolomite filler and PEVAc. Consequently, the tensile strength and elongation at break of the sample also decreased.

The fractured surface of the PEVAc/DNP showed the presence of several tiny voids due to the debonding effect of the filler and the polymer matrix (see Figure 14c). As mentioned earlier, even though their tensile strength increased, it was not as high as the tensile strength of the PEVAc/NP-DNP. This was due to the incompatibility of the DNPs with the non-polar polyethylene molecules of the PEVAc, leading to weak interfacial interactions between the polymer matrix and the filler. The debonding of the dolomite filler and the PEVAc matrix could occur as a tensile load was applied.

The PEVAc/NP-DNP nanocomposite showed a smooth fractured surface with few tiny voids. In one part, there was also an embedded dolomite filler on the surface of the PEVAc matrix (see Figure 14d). In addition, there was a fibrous-like structure that surrounded the embedded dolomite. This showed that the dolomite filler was firmly attached to the PEVAc, as it had higher non-polar PE monomers to interact with the non-polar dolomite. This was reflected by the higher tensile strength achieved by the PEVAc/NP-DNP nanocomposite compared to the other samples. Lim et al. also reported the appearance of a fibrous-like structure inside deformed cavities of a polymer matrix, indicating a strong interfacial interaction between the treated filler and the polymer [17].

## 3. Materials and Methods

### 3.1. Materials

Poly (ethylene-co-vinyl acetate) with 25% vinyl acetate was manufactured by Sigma Aldrich, whereas raw dolomite was supplied by Perlis Dolomite Industries in a powder form with a 150 µm size. This dolomite had a beige color and the chemical formula CaMg(CO_3_)_2_. The stearic acid, with a molecular weight of 284.498 g/mol, was employed as a surface modifier. It had the chemical formula C_18_H_36_O_2_ and a melting range of 66–69 °C. It had a solid white color and a density of 0.847 g/cm^3^. Isopropyl alcohol (2-propanol) was used as a solvent for the stearic acid. Both these chemicals were manufactured by HmbG Chemical and supplied by A.R Alatan Sains Sdn. Bhd. Isopropyl alcohol had the chemical formula C_3_H_8_O, a boiling point of 82.5 °C, and a density of 786 kg/m_3_.

### 3.2. The Preparation of Dolomite Nanoparticles (DNPs) through Physical Modification (Size Reduction)

In the first stage, dolomite was ground by using a Fritsch Pulverisette planetary mill with 50 grinding balls, with a 15 mm diameter and a mass of 13.78 g for each. Three different speeds were used to find the most significant reduction in dolomite size. The speeds that used were 300, 400, and 500 rpm with the same duration of time, 6 h, for each speed. The ratio of the weight of the steel balls to the dolomite powder was 10:1 because the quantity of the material to be milled should not exceed 1/3 of the grinding jar volume. The size reduction in all samples was characterized using a scanning electron microscope (SEM). The parameter of ball milling that gave the greatest size reduction was further used with the sonication method.

In the second part, 10 g of dolomite was dispersed in 100 mL of distilled water. Then, dolomite was sonicated for 2 h with 30% amplitude while the pulse-on value was 10 s and the pulse-off value was 10 s. This sonication method was performed by using a Branson Digital Ultrasonic Disrupter/Homogenizer (Model 450 D). Then, the sample was centrifuged for 10 min at a 4000 rpm speed by using a Rotofix 32 A centrifuge, and the sample was dried at 80 °C for 24 h in an oven before being ground and sieved. This method was repeated with different durations of time, which were 3 and 5 h. All dolomite samples were characterized using an SEM. The parameters of ultrasonication that gave the greatest size reduction were selected and utilized.

**Figure 15:**
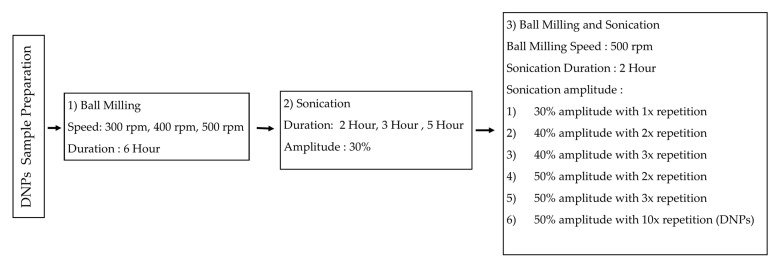
The preparation of dolomite nanoparticles (DNPs).

In the third part, both the ball-milling and ultrasonication parameters that gave the greatest reduction in dolomite size were combined and used. However, different amplitudes and repetition were used in the sonication method. They were (1) 30% amplitude with 1× repetition, (2) 40% amplitude with 2× repetition, (3) 40% amplitude with 3× repetition, (4) 50% amplitude with 2× repetition, (5) 50% amplitude with 3× repetition, and (6) 50% amplitude with 10× repetition. The samples were analyzed using an SEM, TEM, and a particle size analyzer. Figure 15 illustrates the preparation of the dolomite nanoparticles (DNPs). Table 4 summarizes the ball-milling and sonication parameters together with the sample acronyms.

### 3.3. The Preparation of Non-Polar Dolomite Nanoparticles (NP-DNPs) through Chemical Modification (Stearic Acid Treatment)

An amount of 10 g of DNPs was weighed before being added to 100 mL of distilled water. The DNP suspension was stirred for 15 min at 50 °C. Then, 0.16 g of stearic acid was dissolved in 10 mL of isopropyl alcohol at 50 °C. The dissolved stearic acid was added into the dolomite suspension and it was stirred for 3 h by using a homogenizer. The sample was centrifuged for 10 min at 4000 rpm and dried in an oven at 80 °C for 24 h. Finally, the sample was sieved for further use in characterization and as a filler.

### 3.4. The Preparation of PEVAc Nanocomposite

The nanocomposite sample was prepared by using an internal mixer. It was operated at 160 °C and a speed of 36 rpm. The sample was compounded for 10 min. Then, the compounded nanocomposite was compressed into a mold with a 1 mm thickness and a 225 cm^2^ area. It was weighed to 21 g first before being placed into the mold. The sample was compressed by a compression molding machine (GT-7014-H30C). At first, the samples were preheated at 160 °C for 5 min, then pressed for 4 min and cooled for 7 min. Next, the sample was cut according to the testing requirements.

### 3.5. Scanning Electron Microscope (SEM)

**Figure 16:**
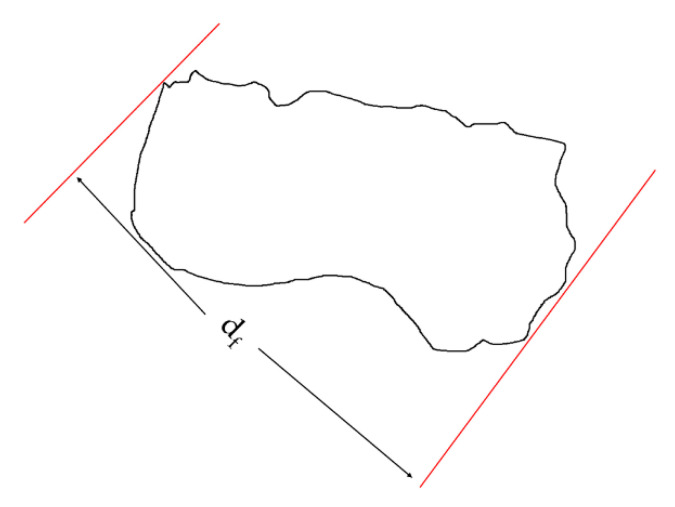
An illustration of the Feret diameter of a particle.

An SEM analysis was performed to characterize the morphology of dolomite, particularly to observe the size reduction of dolomite particles (before and after being milled and sonicated). The virgin PEVAc and PEVAc nanocomposite samples were analyzed based on their fractured surface upon tensile failure. The SEM analysis was performed using a scanning electron microscope (SEM), model SEM-JEOL JSM-6460LA, with a magnification of ×500 and 10 kV of voltage. The size of the dolomite particles was calculated based on the Feret diameter (d_f_) as in Figure 16, and the d_f_ was obtained from imageJ. The Feret diameter refers to the longest distance between any two points along the selected boundary (see Figure 16). This measurement is more suitable for irregularly shaped particles such as dolomite [61].

### 3.6. Transmission Electron Microscopy (TEM)

The size-reduced dolomite particles (D_301x_, D_502x_, D_503x_, and D_510x_ (DNPs)) were analyzed using transmission electron microscopy (TEM), model JEOL JEM2010 (JEOL Ltd. Tokyo, Japan), operating at 200 kV. By using a drop of 2.5 M sucrose solution, the sample was picked up to the 200 mesh Cu grid. Before viewing, the samples were air-dried under a covered petri dish.

### 3.7. Particle Size Analyzer (PSA)

Prior to the particle size analysis, 0.15 mg of dolomite was dispersed in 2 mL of solvent. In order to separate large aggregates and agglomerates in the sedimentation, the dispersion was allowed to stand for 1–2 h. Subsequently, the particle size distribution was measured with a Malvern Instruments Zetasizer Nano ZSP using NIBS technology and dynamic light scattering (Malvern Panalytical Ltd., Malvern, UK).

### 3.8. X-ray Diffractometer (XRD)

A Bruker D2 phaser benchtop X-ray diffractometer (Bruker Corporation, Billerica, MA, USA) was used to characterize changes in the RD, DNPs, and NP-DNPs components. The sample was analyzed in the range of 10° to 55° with a step size of 0.022 and a time per step of 19.2 s. This X-ray diffractometer operated at 30 kV using Cu Ka α rays (λ = 0.15406). The crystallinity or peak-to-noise ratio of the samples was calculated using Equation (1):

(1) $$XRD\;\left(\%\right)=\frac{Ic}{\left(Ic+IA\right)}\times 100\%$$

where *Ic* is the area of the crystalline peaks of the sample, which was obtained by calculating the area under the crystalline peaks, and (*Ic* + *IA*) is the total area under all the peaks of the sample. The crystallite size of the dolomite samples was calculated based on the Scherrer equation, as shown in Equation (2):

L = kλ/β.cosθ(2)

where:

L = crystallite size
k = shape factor
λ= X-ray wavelength (0.154 nm)
β = half-width of the diffraction band (FWHM) (radians)
θ = Bragg angle (peak position in radians)

### 3.9. Fourier-Transform Infrared Spectroscopy (FTIR)

An FTIR analysis was performed to identify and compare the functional groups present in the FTIR spectra of the RD, DNPs, and NP-DNPs. A Perkin Elmer RXI FTIR spectrophotometer (Waltham, MA, USA) was employed for this analysis. The FTIR analysis by the ATR method was performed in the range of 4000–650 cm^−1^. The spectra were recorded with 16 scans and a resolution of 4 cm^−1^.

### 3.10. Contact Angle

A contact angle analysis was performed to analyze the wettability of the dolomite before and after a surface treatment with stearic acid. Dolomite powder was placed onto a thin layer of plasticine on top of a glass slide. It was flattened to obtain a smooth surface. Then, a drop of distilled water was dropped onto the dolomite surface using a syringe. The image of the droplet was captured by a direct phone camera with an attached micro lens. The contact angle was analyzed and calculated using imageJ software with the drop analysis plug-in. Three angles were measured for each sample, and the average value was calculated.

### 3.11. Tensile Analysis

The tensile properties of the virgin PEVAc and the PEVAc nanocomposites were evaluated and compared based on their mean values of tensile strength, their elongation at break, and the modulus of elasticity. The samples were cut according to ASTM D-638-M-5 to obtain dumbbell-shaped samples. Five replicates were prepared for each sample. The tensile test was performed using an Instron machine, model-5582 (Instron^®^, Norwood, MA, USA), with a crosshead speed of 50 mm/min.

## 4. Conclusions

In this research, non-polar dolomite nanoparticles (NP-DNPs) were prepared and used as a nanofiller in a copolymer nanocomposite system. NP-DNPs referred to nano-sized dolomite particles with non-polar properties. By having both a small particle size and non-polar properties, they could easily disperse in the copolymeric matrix, thus serving as an efficient reinforcing filler. To obtain dolomite particles in the nano-size range, raw dolomite was subjected to physical modifications, involving a ball-milling process and tip-ultrasonication procedure. Several ball-milling speeds and tip-sonication parameters were employed, and the best parameters for reducing the size of the dolomite particles to the finest level were identified. The results indicated that the dolomite with the smallest particle size was obtained when it was milled at a speed of 500 rpm and tip-ultrasonicated 10 times at a 50% amplitude. These findings revealed that the combined method of ball milling and tip sonication can produce very fine dolomite particles, down to the nano-size range. Then, NP-DNPs were prepared by treating the DNPs with stearic acid. FTIR and contact angle analyses confirmed the success of this chemical modification. The combination of physical and chemical modifications of the dolomite produced an “easier to disperse” NP-DNP nanofiller for the production of a homogeneous nanocomposite. Thus, this NP-DNP nanofiller was capable of improving the tensile performance of the PEVAc copolymer. The further development of the PEVAc/NP-DNP nanocomposite would allow it to be targeted for more sophisticated applications, such as its use in absorbable materials, biomedical applications, tissue engineering, wound healing, and many more.

## Figures and Tables

**Table 1:** The crystallite size and crystallinity of raw dolomite (RD), dolomite nanoparticles (DNPs), and non-polar dolomite nanoparticles (NP-DNPs).

Samples	Crystallite Size (nm)	Crystallinity (%)	Amorphous (%)
Raw Dolomite (RD)	61.37	83.29	16.71
Polar Dolomite Nanoparticles (DNPs)	57.76	79.07	20.93
Non-Polar Dolomite Nanoparticles (NP-DNPs)	57.12	80.06	19.94

**Table 2:** The average contact angle of raw dolomite (RD), dolomite nanoparticles (DNPs), and non-polar dolomite nanoparticles (NP-DNPs).

Samples	Average Angle (°)
Raw Dolomite (RD)	53.36 ± 0.15
Polar Dolomite Nanoparticles (DNPs)	57.07 ± 0.25
Non-Polar Dolomite Nanoparticles (NP-DNPs)	140.18 ± 1.72

**Table 3:** Tensile properties of raw dolomite (RD), dolomite nanoparticles (DNPs), and non-polar dolomite nanoparticles (NP-DNPs).

Sample	Tensile Strength (MPa)	Elongation at Break (%)	Modulus of Elasticity (MPa)
PEVAc	7.87 ± 0.11	1107.73 ± 22.60	1.17 ± 0.058
PEVAc/RD	7.12 ± 0.20	1089.33 ± 18.43	1.03 ± 0.058
PEVAc/P-DNP	8.30 ± 0.43	1157.63 ± 23.71	0.77 ± 0.058
PEVAc/NP-DNP	8.76 ± 0.31	1169.90 ± 8.6	0.73 ± 0.058

**Table 4:** Ball-milling and sonication parameters for dolomite samples.

Type of Dolomite Sample	Speed (rpm)	Duration (h)	Sonication Duration (h)	Sonication Amplitude (%)	No. of Tip Sonication Repetitions	Sample Acronyms	
Raw dolomite	-	-	-	-	-	RD	
Ball milling	300	6	-	-	-	D_300_	
	400	6	-	-	-	D_400_	
	500	6	-	-	-	D_500_	
Ultrasonication	-	-	1	30	1	D_301hr_	
	-	-	2	30	1	D_302hr_	
	-	-	5	30	1	D_305hr_	
Ball milling + tip-sonication	500	6	2	30	1	D_301x_	
	500	6	2	40	2	D_402x_	
	500	6	2	40	3	D_403x_	
	500	6	2	50	2	D_502x_	
	500	6	2	50	3	D_503x_	
	500	6	2	50	10	D_5010x_	

## Data Availability

There are no linked research datasets for this submission. Data will be made available on request.
